# Structural Characterization of the Extracellular Polysaccharide from *Vibrio cholerae* O1 El-Tor

**DOI:** 10.1371/journal.pone.0086751

**Published:** 2014-01-24

**Authors:** Fitnat Yildiz, Jiunn Fong, Irina Sadovskaya, Thierry Grard, Evgeny Vinogradov

**Affiliations:** 1 Department of Microbiology and Environmental Toxicology, University of California Santa Cruz, Santa Cruz, California, United States of America; 2 National Research Council, Ottawa, Ontario, Canada; 3 Université du Littoral-Côte d’Opale, Boulogne-sur-mer, France; Université d’Auvergne Clermont 1, France

## Abstract

The ability to form biofilms is important for environmental survival, transmission, and infectivity of *Vibrio cholerae*, the causative agent of cholera in humans. To form biofilms, *V. cholerae* produces an extracellular matrix composed of proteins, nucleic acids and a glycoconjugate, termed *Vibrio* exopolysaccharide (VPS). Here, we present the data on isolation and characterization of the polysaccharide part of the VPS (VPS-PS), which has the following structure: 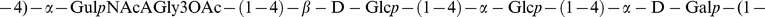
where α-D-Glc is partially (∼20%) replaced with α-D-GlcNAc. α-GulNAcAGly is an amide between 2-acetamido-2-deoxy-α-guluronic acid and glycine. Apparently, the polysaccharide is bound to a yet unidentified component, which gives it high viscosity and completely suppresses any NMR signals belonging to the sugar chains of the VPS. The only reliable method to remove this component at present is a treatment of the whole glycoconjugate with concentrated hydrochloric acid.

## Introduction

The facultative human pathogen *Vibrio cholerae* is responsible for cholera, a significant disease in developing countries and areas impacted by natural and man-made disasters [1], [2]. *V. cholerae* is able to form biofilms - *i.e.*, matrix-enclosed surface-associated communities, which is critical for it environmental survival, transmission and infectivity [3], [4]. In aquatic environments, *V. cholerae* forms biofilms on surfaces of phytoplankton, zooplankton, aquatic plants, crustaceans, and insects [5]–[9]. In addition, surface waters of cholera endemic areas harbor *V. cholerae* as clumps or biofilm-like aggregates, and removal of particles >20 µm in diameter from water can decrease cholera incidence by half [10], [11]. It is well accepted that biofilm formation in aquatic ecosystems both enhances survival and persistence of *V. cholerae,* by providing nutrients and protection from protozoan grazing and phage predation [12], and that it plays a critical role in the transmission of the pathogen.

*V. cholerae* also forms biofilms while inside infected individuals. Stool samples from cholera patients contain *V. cholerae* both in biofilm-like clumps and in a planktonic form [4]. The average infectivity of biofilm-like clumps is higher than that of planktonic cells, and growth in biofilm induces a hyper-infectious phenotype, suggesting that *V. cholerae* biofilms are important in the disease process [4], [13].

Biofilm formation depends on production of a biofilm matrix *i.e.*, exopolysaccharides, proteins, and nucleic acids [14]. In many bacteria exopolysaccharides represent a major portion of the biofilm matrix; and mutants unable to produce exopolysaccharides are impaired in biofilm formation [15]. Forexample, the opportunictics human pathogen *Pseudomonas aeruginosa* has the capacity to produce several polysaccharides that contribute to biofilm formation. One of them is alginate, produced by mucoid *P. aeruginosa* strains isolated from patients with cystic fibrosis. Alginate is a partially O-acetylated unbranched heteropolymer of β- 1–4 linked D-mannuronic acid and L-guluronic acid residues. *P. aeruginosa* strains with enhanced alginate production form biofilms that have highly structured architecture,and are resistant to the antibiotic tobramycin [16]. Non-mucoid *P. aeruginosa* strains produce Psl and/or Pel. Psl polysaccaride is consist of a branched pentasaccharide repeating unit composed of D-mannose, D-glucose and L-rhamnose residues [17]. Pel is a glucose-rich polysaccharide of unknown structure [18]. Pel polysaccharide is involved in maintaining cell-cell interactions in biofilms, while Psl appears to be the primary structural polysaccharide for maintenance of mature biofilm architecture [19].

Polysaccharide intercellular adhesin (PIA) or polymeric N-acetyl-glucosamine (PNAG), consisting of partially N-deacylated poly-1,6-β-GlcNAc, plays critical roles in *Staphylococcus aureus* and *Staphylococcus epidermidis* biofilm development [20], [21]. PIA-like polymers also serve as adhesins that stabilize biofilms of *E. coli* [22].

The importance of exopolysaccharides in biofilm formation has also been recognized in Vibrio spp. *Vibrio fischeri* uses symbiosis polysaccharide biofilms,Syp, to colonize its host, the squid *Euprymna scolopes* [23]. *V. parahaemolyticus* polysaccharide CPSA contains approximately equal amounts of fucose, galactose, glucose and N-acetylglucosamine and is critical for biofilm formation [24]. Structures of exopolysaccharides produced by these Vibrio species remains to be determined.

One of the major components of *V. cholerae* biofilm matrix is *Vibrio* exopolysaccharide (VPS) [25], [26]. The VPS biosynthesis genes are found in two regions on the large chromosome of *V. cholerae* O1 El Tor [*vps*U (VC0916), *vpsA-K*, VC0917-27 (*vps-*I cluster); *vpsL-Q*, VC0934-9 (*vps-*II cluster)] [26]. Systematic deletion of *vps* genes and phenotypic analysis of *vps* mutants has revealed that most of the genes of the *vps* clusters are required for VPS biosynthesis and biofilm formation [26]. Recent studies using a rabbit ileal loop model system have revealed that VPS is required for *in vivo* biofilm formation [27]. Infant mouse colonization assays have also revealed that strains unable to produce VPS exhibit a defect in intestinal colonization compared to the wild-type [26]. Taken together, these studies highlight the importance of the biofilm growth mode and VPS production in both the intestinal and transmission phases of *V. cholerae*’s life cycle.

We previoulsy reported composition and linkage analysis of the crude VPS preparations from rugose variant of *V. cholerae* O1 El Tor [25]. However, the precise chemical structure of VPS was unknown. We report here, for the first time, the chemical structure of the polysaccharide part of VPS, produced by the *V. cholerae* O1 El Tor rugose variant, A1552R, secreting high levels of VPS [25]. Characterization of the major structural component of *V. cholerae* biofilm matrix sets the stage for further structure-function analysis of the biofilm matrix and will facilitate identification of targets to combat this deadly pathogen.

## Materials and Methods

### Bacterial Strains and Growth Conditions

Cells of the *Vibrio cholerae* rugose variant, A1552R (FY_VC_2), which produces high level of VPS [26] were routinely grown in Luria-Bertani (LB) medium (1% tryptone, 0.5% yeast extract, 1% NaCl) at 30°C.

To isolate biofilm matrix, overnight-grown cultures were spread on cellulose dialysis membranes placed on the surface of M9 agar plates (150×15 mm) supplemented with glucose (10 mM), casamino acids (0.5%) and MEM vitamins and incubated for 48 h at 30°C. Bacteria from these plates are harvested and suspended in phosphate-buffered saline (PBS). The samples are shaken on a rotary shaker at 4°C for 24 h. During this time, the biofilm matrix separates from the cells and crude biofilm matrix was isolated and processed as described below.

### Preparation of the VPS

Biofilm cells were collected by centrifugation (5,000g, 4°C, 45 min). The supernatant was clarified with additional centrifugation (8,000×g, 4°C, 45 min) and dialyzed for 2 days against distilled water containing 0.02% sodium azide. We used cellulose tubing MWCO 12–14 kDa, with repeated change of the water The dialyzed sample was lyophilized to give crude VPS extract (typical yield was 400 mg per 100 plates). This material was dissolved in water at about 1 mg/mL (it dissolves slowly, and the solution is viscous and turbid) and treated with DNAse, RNAse (37°C, 24 h), and then Proteinase K (pH 8 by TRIS-HCl buffer, 37°C, 48 h) lipopolysaccharide (LPS) was then removed by ultracentrifugation at 150 000 g for 3 h, solution dialyzed against water for 3 days, to give clear and not very viscous solution, which was lyophilized to give VPS. The yield from the material after enzymatic treatment was about 50%.

### Preparation of the Polysaccharide Part of the VPS (VPS-PS)

VPS (50 mg) was dissolved in 37% HCl (2 mL) to give the clear solution a violet color. It was kept for 15 min at room temperature, poured into 100 mL of water and lyophilized. Dried material was washed with water; the precipitate was removed by centrifugation; and the solution passed through a Biogel P6 column to produce polymeric material. This contained VPS-PS and a small amount of the O-polysaccharide, which was removed by anion-exchange chromatography on Hitrap Q column in a gradient of NaCl from 0 to 1 M in 1 h. The VPS-PS was eluted as a broad peak over 10 min and collected in 3 parts, each of which had the same purity and structure. The yield of the VPS-PS from VPS was about 50–70%. A small amount of water-insoluble material was obtained, which we were able to partially dissolve in chloroform, with the rest soluble in DMSO.

### General and Analytical Methods

NMR experiments were carried out on a Varian INOVA 500 MHz (^1^H) spectrometer with 3 mm gradient probe at 25–50°C with acetone internal reference (2.225 ppm for ^1^H and 31.45 ppm for ^13^C), using standard pulse sequences gCOSY (gradient COrrelation SpectroscopY), TOCSY (Total Correlation Spectroscopy) (mixing time 120 ms), NOESY (Nuclear Overhauser Effect Spectroscopy) (mixing time 300 ms), gHSQC (gradient Heteronuclear Single Quantum Coherence), and gHMBC (gradient Heteronuclear Multiple Bond Coherence) (100 ms long range transfer delay). AQ time was kept at 0.8–1 sec for H-H correlations and 0.25 sec for HSQC. 256 increments were acquired for t1 in all 2D spectra, except 512 for gCOSY.

### Chromatography

Gel chromatography was performed on a Sephadex G-15 column (1.5×60 cm) or a Biogel P6 column (2.5×60 cm) in pyridine-acetic acid buffer (4 mL:10 mL:1 L water), and monitored by refractive index detector (Gilson). Anion exchange chromatography was done on an Hitrap Q column (2×5 mL size, Amersham), with UV monitoring at 220 nm in a linear gradient of NaCl (0–1 M, 1 h) at the 3 mL/min. Fractions of 1 min were collected and additionally tested for carbohydrates, by spotting on an SiO_2_ TLC plate, dipping them in 5% H_2_SO_4_ in EtOH and heating with a heat-gun. All fractions of interest were dried in a Savant drying centrifuge and ^1^H spectra were recorded for each fraction without desalting. For 2D NMR, desalting was performed on a Sephadex G15 column.

### Monosaccharide Analysis

Samples with added inositol standard were hydrolyzed with 3 M TFA at 120°C. Monosaccharides were converted to alditol acetates by conventional methods and identified by GC-MS on a Trace GC Ultra apparatus, coupled with a DSQ II Mass spectrometer (Thermo Scientific), and equipped with a TR-5MS capillary column (30 m×0.25 mm ID×0.25 µm film) with helium carrier gas, using a temperature gradient 170°C (3 min), 250°C at 5°C·min^−1^.

### Methanolysis

A sample (<0.5 mg) in methanol (0.5 mL) was cooled in dry ice and 0.05 mL of acetyl chloride was added, the vial closed and heated at 90°C for 3 h, dried and acetylated with 0.3 mL Ac_2_O - 0.3 mL pyridine (30 min, 90°C), dried again using a stream of air, and analyzed by GC-MS on a Varian Saturn 2000 instrument (EI-ion trap) on a DB-17 capillary column, at a temperature gradient of 170–270°C at 4°C/min.

### Determination of Absolute Configurations of Monosaccharides

To the polysaccharide sample (0.2 mg) (*R*)-2-BuOH (0.2 mL) and acetyl chloride (0.02 mL) were added at room temperature, heated at 90°C for 2 h, dried by air stream, acetylated, analyzed by GC-MS as described above. Standards were prepared from monosaccharides of known configuration with (*R*)- and (*S*)-2-BuOH.

### Periodate Oxidation

VPS-PS (10 mg) was dissolved in water (2 mL) and NaIO_4_ (20 mg) was added. This solution was kept for 24 h. Ethylene glycol (0.2 mL) and the excess NaBD_4_ were added, with the solution kept for 1 h, treated with 0.2 mL of AcOH, and desalted on a Sephadex G-15 column. The product was hydrolyzed with 2% AcOH 2 h at 100°C, and desalted on a Sephadex G-15 column to give OS1.

## Results

### Preparation and Properties of the VPS and its Polysaccharide Part, VPS-PS

VPS was first identified and characterized in a rugose variant of *V. cholerae* A1552R [25]. This rugose variant has enhanced capacity to produce VPS and we have utilized A1552R to gain a better understanding of the chemical structure of the VPS.

**Figure 1:**
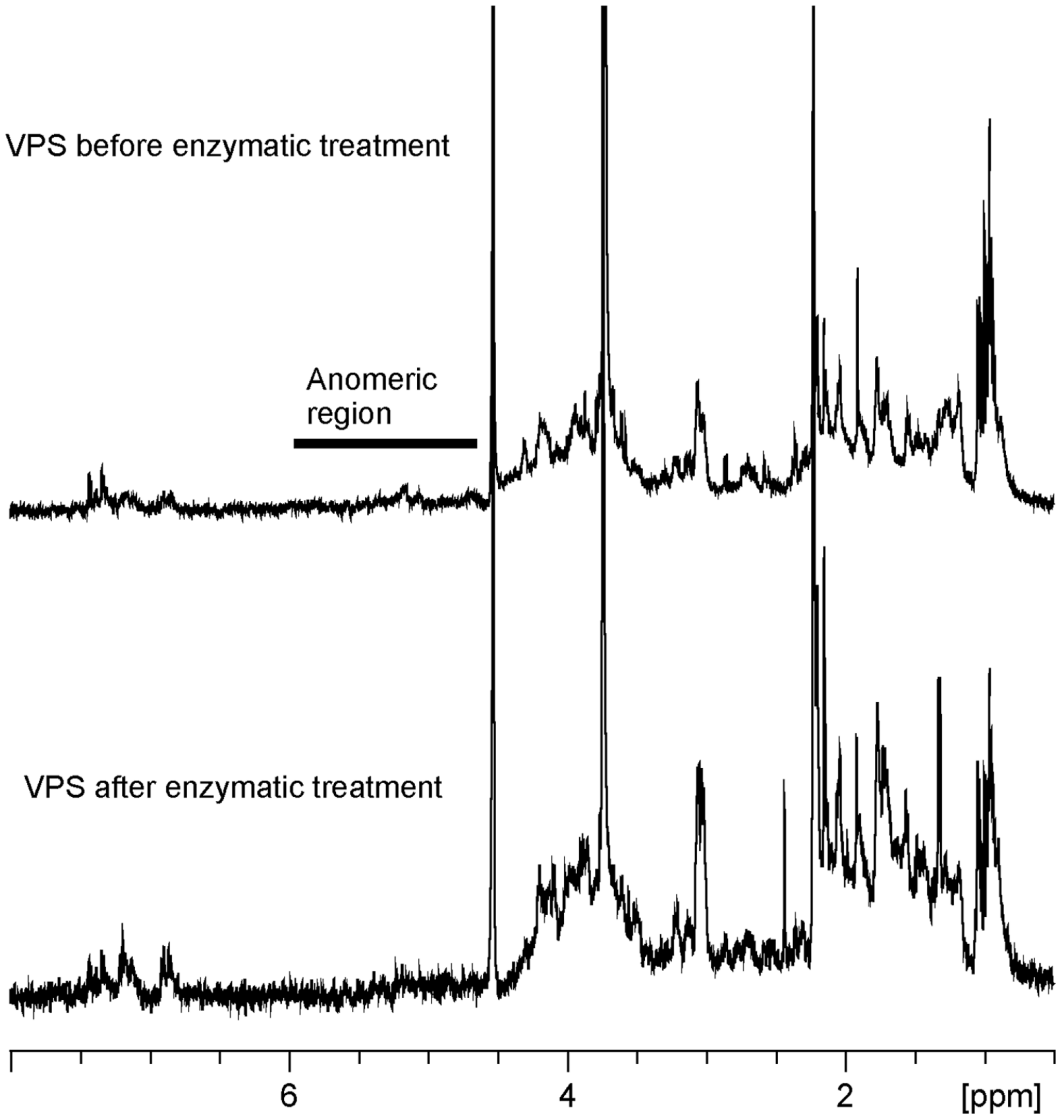
^1^H NMR spectra of the VPS before and after treatment with enzymes (50°C).

The *Vibrio cholerae* rugose variant and an isogenic strain lacking *vps* gene clusters *vps*-I and *vps*-II (Δ-VPS) were grown in a chemically defined medium which did not contain HMW carbohydrates. The crude extracellular (EC) material was recovered from the biofilm matrix. Aqueous solutions of the crude EC material of the rugose strain were very viscous, 100 mg of the material dissolved in 50 mL of water produced gel-like solution. The viscosity of a corresponding sample from the Δ-VPS strain was much less pronounced. Crude r-VPS had a higher carbohydrate/protein ratio than the corresponding Δ-VPS sample (∼20 and ∼2 µg carbohydrate per 1 µg of protein (in Glc/BSA equivalents), respectively). UV measurements (260 and 280 nm) of the non-dialyzable extracellular material indicated that the protein-nucleic acid content was about 30%. NMR spectra of the whole high-molecular mass extracellular material showed signals characteristic for proteins, lipids, and nucleic acids. No significant signals of the anomeric protons of sugars (4.5–6 ppm) were observed in ^1^H NMR spectra at neutral, acidic or basic pH (Fig. 1). Monosaccharide analysis showed the presence of ∼20% glucose and 10–15% galactose, as well as small amounts of LD-heptose (indicating the presence of LPS), glucosamine, and perosamine. To remove nucleic acids and proteins, a dialyzed extracellular material was treated with DNAse, RNAse, and then Proteinase K. LPS was removed by ultracentrifugation, the transparent and colorless solution was dialyzed and lyophilized, with the overall yield of the VPS at about 50 to 80% of the starting crude material.

The resulting VPS material showed no sugar signals in ^1^H NMR spectrum in D_2_O at temperatures up to 50°C (Fig. 1). NMR spectra of the whole high-molecular mass extracellular material contained a complex mixture of signals, but clearly did not include sugar anomeric signals. The DMSO solution was extremely viscous, and also showed no meaningful NMR signals.

Several methods (ultracentrifugation, gel chromatography, anion-exchange chromatography, mild acid or base hydrolysis, 48% HF, EDTA treatment) were tried in order to isolate the polysaccharide part of the VPS so that it could be used for further analysis, all with no success. Alkaline treatment produced a small amount of partially depolymerized VPS-PS (polysaccharide part of VPS), which was initally used for the structural analysis. Occasionally, it was found that treatment of the VPS with 37% HCl at room temperature for a short time releases a polysaccharide VPS-PS, suitable for NMR analysis.

VPS-PS was additionally purified by anion-exchange chromatography to remove minor impurities of the typical *V. cholerae* O1 LPS O-chain. VPS-PS was eluted along a NaCl gradient, resulting in several broad peaks. Three fractions were collected. Each of the fractions had identical NMR spectra, and probably differed in molecular mass. Monosaccharide analysis of the VPS-PS showed the presence of glucose, galactose, and a smaller amount of glucosamine. All these monosaccharides had a D-configuration, determined by GC of 2-butyl glycoside acetates.

**Figure 2:**
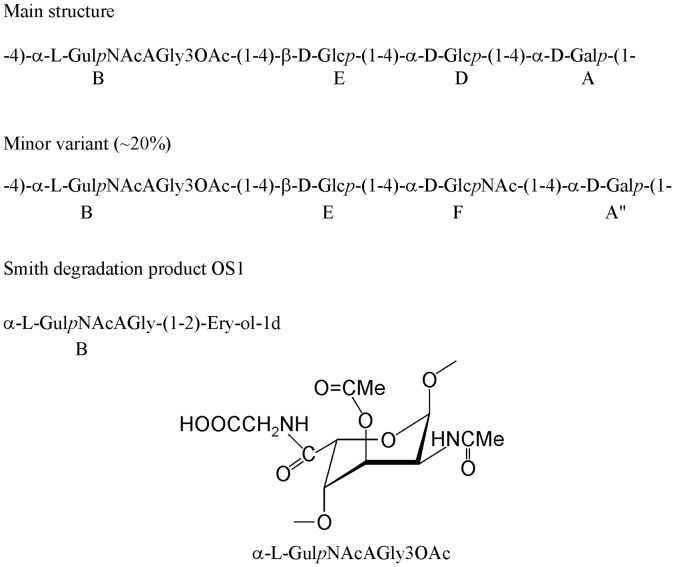
Structures of the VPS-PS and its derivative.

**Figure 3:**
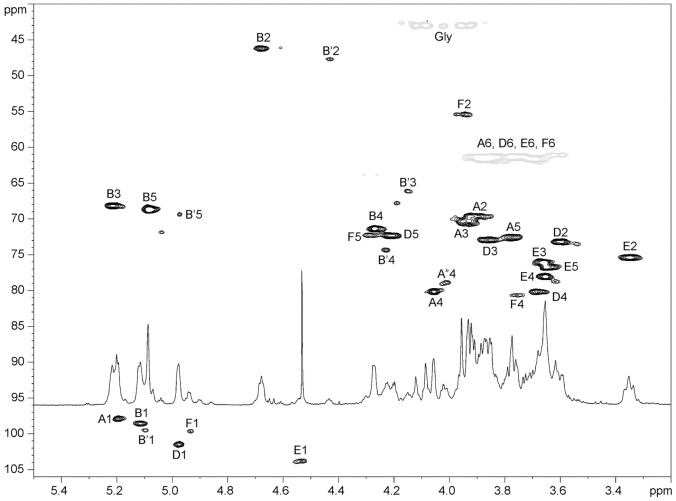
^1^H-^13^C HSQC spectrum of the VPS-PS with ^1^H NMR trace.

**Figure 4:**
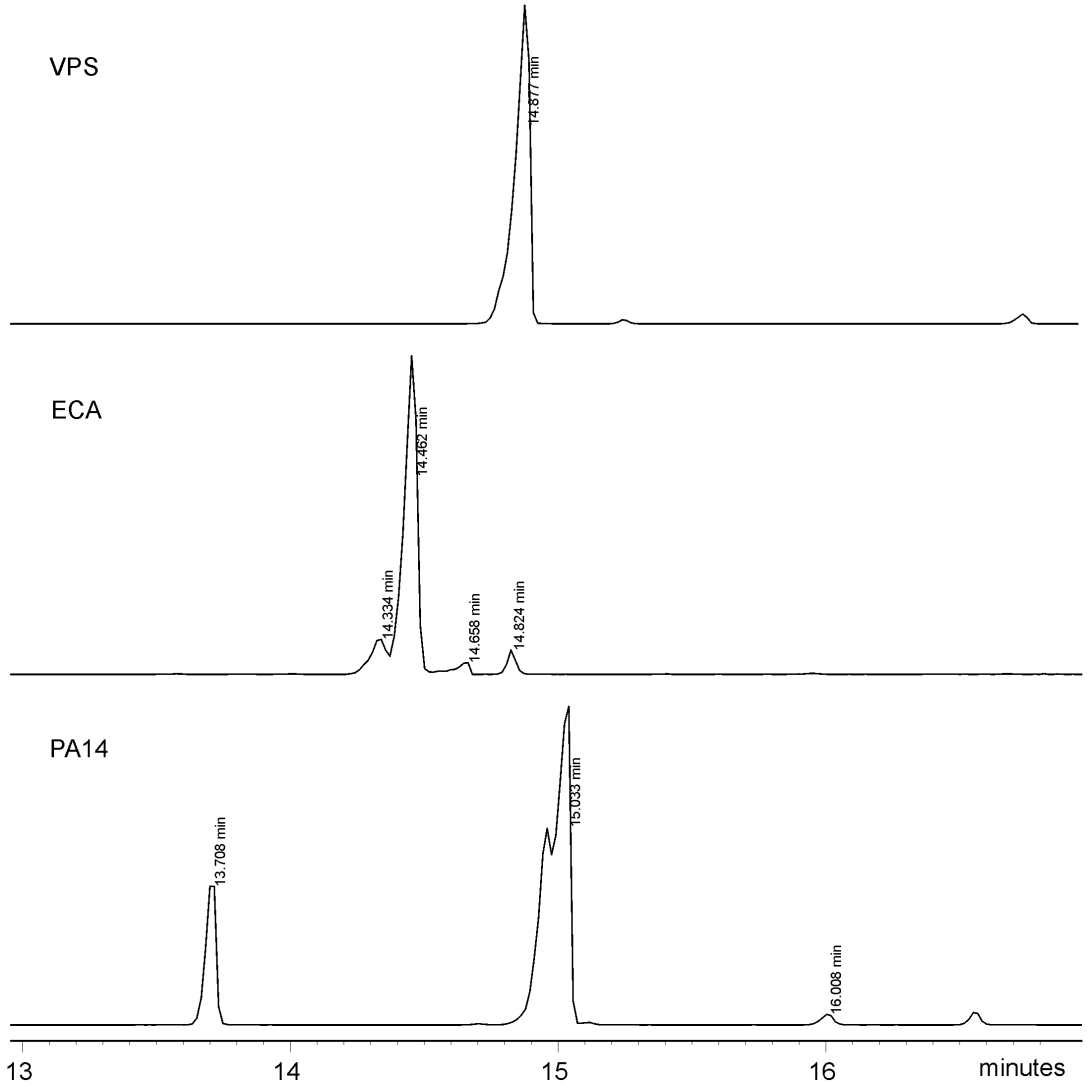
GC-MS profiles of acetylated methanolysis products selected for m/z 316 (oxonium ion from hexosaminouronic acid) of VPS-PS, ECA (ManNAcA), and O-PS from *P. aeruginosa* PA14 (GalNAcA).

**Figure 5:**
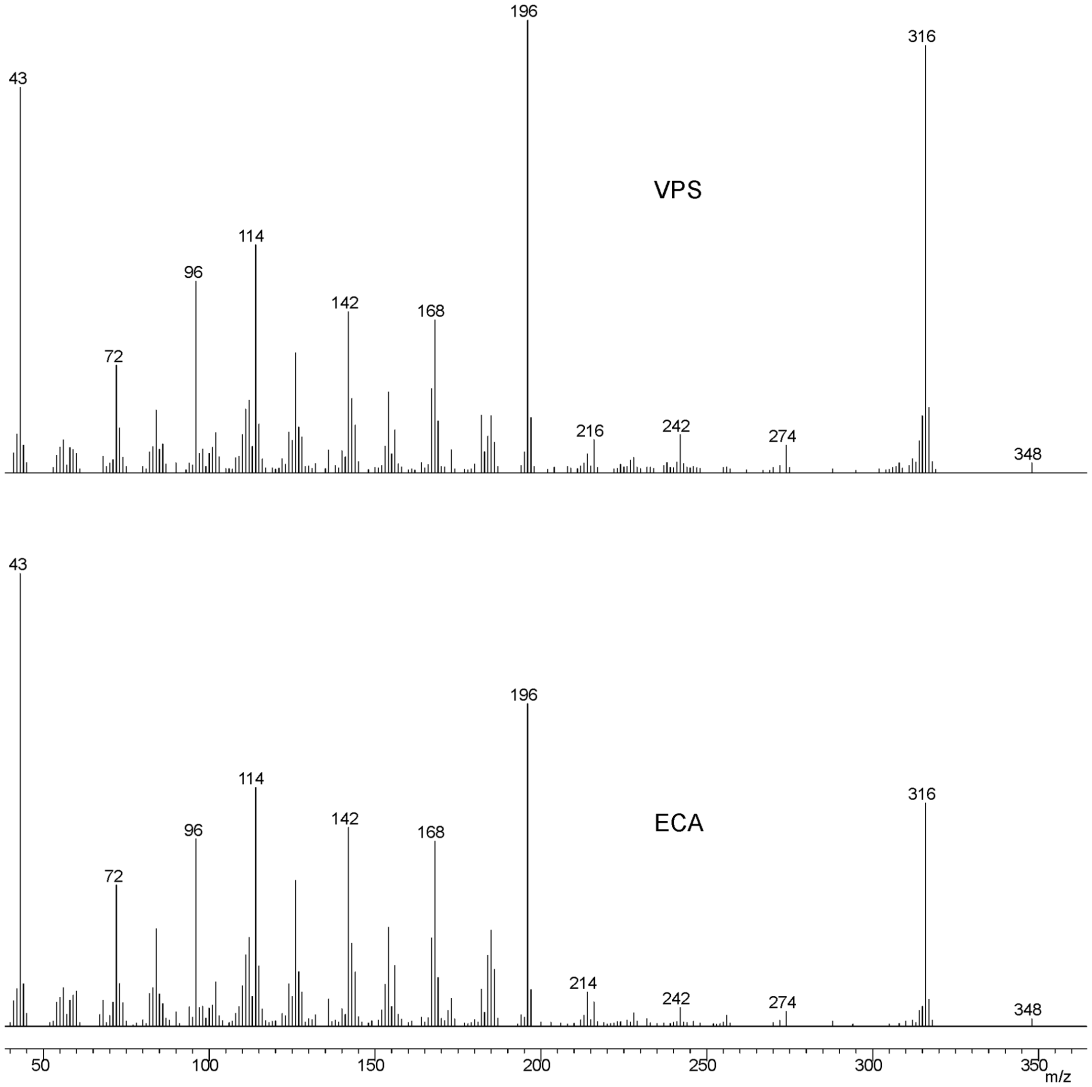
Mass spectra of acetylated methyl glycosides methyl esters of 2-aminouronic acids obtained by methanolysis from VPS-PS and ECA (contains ManNAcA).

VPS-PS structure was analyzed using 2D NMR spectroscopy. Four major monosaccharide spin-systems were identified in the spectra. Three of them belonged to α-Gal, α-Glc and β-Glc (Figs. 1–3, Table 1). The fourth monosaccharide had an aminogroup at position 2 (C-2 signal at 46.1 ppm) and no H-6 protons. In HMBC spectrum its H-4 and H-5 showed correlations with a carbonyl carbon signal (Table 1), indicating that it was an uronic acid. Intraring vicinal proton coupling constants were all <3 Hz in this monosaccharide. High-field position of the C-2 signal together with all small proton couplings indicated that it was a 2-acetamido-2-deoxy-α-guluronic acid. There was no standard available for the direct identification of this monosaccharide, however, it was shown to be different from mannosaminouronic acid (from enterobacterial common antigen, ECA, [28]) and galactosaminouronic acid (from *P. aeruginosa* PA14 O-specific polysaccharide [29]) by GC of acetylated methanolysis products (Fig. 4). Mass spectra of the derivatives of all aminouronic acids were identical (Fig. 5).

**Table 1:** ^1^H and ^13^C chemical shifts of the VPS-PS and its derivatives.

		H/C-1	H/C-2	H/C-3	H/C-4	H/C-5	H/C-6
α-Gal **A**	H	5.19	3.91	3.93	4.06	3.77	3.77; 3.77
	C	97.9	69.6	70.3	80.2	72.5	61.4
α-Gal **A″**	H	5.20	3.91	3.98	4.01	3.77	3.77; 3.77
	C	99.5	69.6	70.0	78.8	72.5	61.4
**B**	H	5.11	4.68	5.21	4.27	5.08	
	C	98.5	46.1	68.2	71.3	68.6	172.2
O-Deacylated **B**	H	5.09	4.44	4.15	4.22	5.10	
	C	99.0	47.1	65.6	73.9	67.8	
α-GlcNAc **F**	H	4.93	3.95	3.93	3.76	4.28	3.84; 3.91
	C	99.6	55.4	70.3	80.6	72.2	61.4
α-Glc **D**	H	4.97	3.60	3.86	3.68	4.21	3.85; 3.89
	C	101.5	73.2	72.9	80.1	72.3	61.4
β-Glc **E**	H	4.53	3.35	3.66	3.65	3.64	3.70; 3.87
	C	103.8	75.4	76.1	78.1	76.7	61.5
Gly	H		3.94; 4.10				
	C	175.5	43.0				
**B, OS1**	H	5.18	4.34	3.97	4.18	4.82	
	C	99.3	47.2	69.9	70.5	68.3	
Gly, **OS1**	H		3.91; 3.99				
	C		43.4				

Spectra of the polysaccharide were recorded at 500 MHz, at 50°C in D_2_O, OS1 at 25°C. Residue A″ belongs to the structure with GlcNAc F, instead of Glc D. NAc signal in VPS-PS at 2.04/23.3 ppm; OAc at 2.19/22.0 ppm (H/C).

The H-3 signal of the aminoguluronic acid residue B was shifted to the low-field (5.21 ppm), due to the O-acetylation at O-3. A minor variant of the non-O-acetylated residue B was also observed in the spectra. The O-acetyl signal was observed at 2.19/22.0 ppm (H/C).

The HSQC spectrum of the VPS-PS contained a set of non-sugar signals at 3.9–4 ppm (H)/43.4 ppm (C), correlating to a carbonyl signal at 175.5 ppm. Position of these signals indicate a glycine spin system. Glycine was linked to GulNAcA by the amide linkage between GulNAcA C-6 and Gly N-2, which was confirmed by the observation of the HMBC correlation between Gly H-2 and GulNAcA C-6 at 172.2 ppm. The presence of an amide was also confirmed by the recording of the HSQC spectra at different pH (3, 5 and 9), which showed that GulNAcA signals were not sensitive to acidity and, consequently, its carboxyl group cannot ionize due to amidation.

The sequence of the monosaccharides was determined using NOESY data, where the following correlations were observed: A1:B4; B1:E3,4,5; D1:A4; E1:D4, indicating the sequence as shown on Fig. 2. The β-Glc residue E had overlapping protons 3,4,5; thus, NOE or HMBC could not be used to determine its substitution. Selection between possible substitution at O-3 or O-4 was based on the fact that a non-substituted C-4 signal of β-Glc should be around 70 ppm, and there was no such signal in the spectra. Consequently, β-Glc E was substituted at O-4.

**Figure 6:**
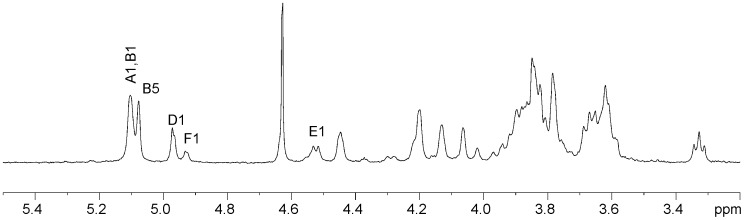
^1^H NMR spectrum of the O-deacetylated VPS-PS.

The ^1^H NMR spectra of VPS-PS contained several small signals in the anomeric region (Fig. 3). Also, several smaller O-and N-acetate signals were visible (not shown). Deacylation of the VPS-PS with ammonia (24% NH_4_OH, 25°C, 16 h) led to the disappearance of nearly all minor signals (Fig. 6). However, some signals remained and were identified as belonging to a structural variant, in which α-Glc D was replaced by α-GlcNAc F. This variant constituted about 20% of the total structure.

**Figure 7:**
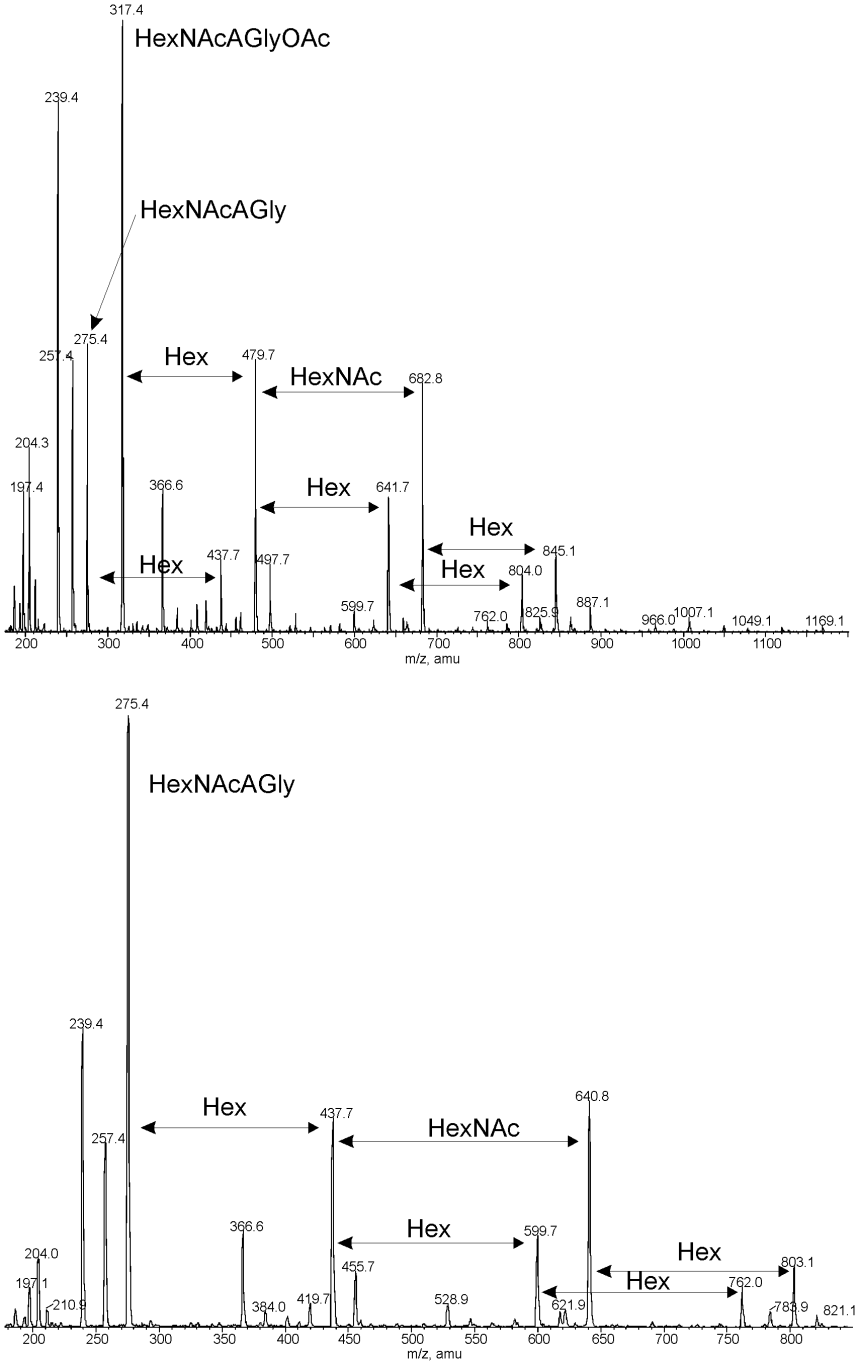
Positive mode MS-MS spectra of the VPS-PS (top) and O-deacylated VPS-PS (bottom).

Positive and negative mode ESI-MS analysis of the whole VPS-PS showed no peaks, indicating the polymeric nature of VPS-PS. In the high orifice voltage MS-MS spectrum of VPS-PS (Fig. 7) a major peak of HexNAcAGlyOAc at m/z 317.4 was observed. A series of higher mass signals was observed which corresponded to an addition to HexNAcAGlyOAc of a hexose, and then another hexose or N-acetylhexosamine (corresponding to variants with Glc D or GlcNAc F), continuing with another hexose. The same sequence was observed in the MS-MS spectrum of the O-deacylated VPS-PS, except that it lacked the O-acetate (HexNAcAGly at m/z 275.4).

Periodate oxidation of the VPS-PS produced a glycoside OS1 (Fig. 2), containing α-GulNAcAGly and erythritol (NMR data in Table 1). O-Acetate was mostly lost during the treatment.

Assignment of the signals was confirmed by ^13^C spectra calculations [30], with α-D-GalNAcA or α-L-GalNAcA used, instead of α-GulNAcA. Chemical shifts for units A, D, and E showed good agreement with experimental data when α-L-GalNAcA was used, except for C-1 of α-Gal, which had an experimental chemical shift of 97.9 ppm and calculated chemical shift at 100.1 ppm (2.2 ppm deviation). With the use of α-D-GalNAcA for calculation this deviation increased to 3.6 ppm. Furthermore, the use of α-D-GalNAcA produced a large error for E-4, which was 2 ppm off. This points to a most probable L-configuration of GulNAcA, which would be also expected considering possible biosynthesis of L-GulNAcA from D-ManNAcA by inversion of its C-5.

Cleavage of the VPS using concentrated HCl produced a minor amount of the precipitate, which was partially dissolved in chloroform and the rest in DMSO. However, NMR spectra of these compounds were not informative. They contained large signals of fatty acids and messy small signals elsewhere. GC analysis of methanolysis products of the precipitate led to the identification of C16, Δ-C16, C18, and Δ-C18 acids. Acetylated methanolysate contained additionally derivatives of the monosaccharides found in VPS-PS, but no acetylated hydroxy-fatty acids. MALDI mass spectrum of the precipitate showed two major peaks at m/z 730 and 758. The mass difference of 28 indicated that signals belong to a lipid (2×CH_2_ extension of fatty acids, due to the replacement of C16 to C18). The nature of this lipid remains unknown.

## Discussion

*V. cholerae* forms biofilms during aquatic and intestinal stages of its life cycles. Biofilms gain their structure and integrity, in part, from the exopolysaccharide VPS. We have previously isolated VPS, performed initial glycosyl composition and linkage analyses, and identified the gene clusters required for VPS biosynthesis. However, chemical structure of VPS has remained elusive due to difficulties in obtaining its NMR spectra. In this study, we report the chemical structure of VPS, and describe a reliable method for the isolation of VPS from the extracellular material produced by *V. cholerae*.

While *V. cholerae* biofilm matrix contains proteins, nucleic acids, lipids, and LPS, VPS-PS is clearly its main component, constituting more than 50% of the material by mass. Lipids released from VPS by HCl treatment were obtained in small amounts. VPS-PS showed no signals in the NMR spectra, until its release with HCl. What actually happens during HCl treatment remains unclear, as well as the nature of the non-carbohydrate part of the VPS and its bond to the VPS-PS. All fractions of size-exclusion chromatography separation of HCl-treatment products were tested by NMR and no components that could be released from the VPS-PS was found. VPS-PS spectra contained no visible signals of reducing monosaccharides, thus it was not significantly depolymerized. Although it seems a harsh treatment, concentrated HCl caused no observable degradation of the polysaccharide.

VPS-PS contains a novel monosaccharide, 2-acetamido-2-deoxy-L-guluronic acid, which is present in the polymer as its amide with glycine - another unusual component of natural polysaccharides. 2-Acetamido-2-deoxy-L-guluronic acid could be biosynthesized from 2-acetamido-2-deoxy-D-mannuronic acid by the enzymatic inversion of C-5 configuration, like it happens in the conversion of D-mannuronic acid into L-guluronic acid in alginate biosynthesis [31], or D-glucuronic conversion to L-iduronic in heparin-like polymers [32]. Interestingly, one of the *vps* gene cluster genes (*vpsB*/VC0918) encodes a protein predicted to function as UDP-glucose/GDP-mannose dehydrogenase, which catalyzes formation of UDP-glucuronic acid or GDP-mannuronic acid. This is in good agreement with the presence of the acidic sugar GulNAcA in the VPS structure. Two genes involved in *vps* biosynthesis operon - *vpsC* (VC0919) and *vpsG* (VC0923) - are predicted to encode serine acetyltransferase-related proteins that may be involved in VPS O-acetylation. The presence of GlcNAc is consistent with our previous report showing that VPS could be stained with a Cy3-labeled wheat germ agglutinin, which recognizes GlcNAc [33].

Different structures of capsular polysaccharides have been reported for two strains of *V. cholerae*: O139 ([34]–[36] and NRT36S [37]. Of these two examples, O139 CPS consists of the components of the LPS O-chain, but NRTC36S CPS has some properties common with VPS-PS, in that it contains uronic acids and is O-acetylated.

Humans ingest *V. cholerae* biofilms as part of the pathogen’s normal transmission route. The presence of the VPS in these biofilms may influence progression of the disease, and/or development of an immune response against *V. cholerae*. Thus, characterization of the complete chemical structure of VPS and better understanding of VPS biosynthesis could lead to development of inhibitors of biofilm formation and, thus, reduce transmission of the pathogen.
